# Root trait diversity, molecular marker diversity, and trait-marker associations in a core collection of *Lupinus angustifolius*

**DOI:** 10.1093/jxb/erw127

**Published:** 2016-04-05

**Authors:** Yinglong Chen, Fucheng Shan, Matthew N Nelson, Kadambot HM Siddique, Zed Rengel

**Affiliations:** ^1^School of Earth and Environment, The University of Western Australia, Perth, WA 6009, Australia; ^2^The UWA Institute of Agriculture, The University of Western Australia, Perth, WA 6009, Australia; ^3^The State Key Laboratory of Soil Erosion and Dryland Farming on the Loess Plateau, Northwest A&F University, and Chinese Academy of Sciences, Yangling, Shaanxi 712100, China; ^4^The Department of Agriculture and Food, Western Australia, Locked Bag 4, Bentley, WA 6983, Australia; ^5^School of Plant Biology, The University of Western Australia, Perth, WA 6009, Australia; ^6^ Current address: Natural Capital and Plant Health, Royal Botanic Gardens Kew, Wakehurst Place, Ardingly, West Sussex, RH17 6TN, UK

**Keywords:** Diversity Arrays Technology (DArT), genetic diversity, molecular marker, narrow-leafed lupin, root trait, trait-marker association.

## Abstract

Narrow-leafed lupin (*Lupinus angustifolius* L.) is the predominant grain legume crop in southern Australia, contributing half of the total grain legume production of Australia. Its yield in Australia is hampered by a range of subsoil constraints. The adaptation of lupin genotypes to subsoil constraints may be improved by selecting for optimal root traits from new and exotic germplasm sources. We assessed root trait diversity and genetic diversity of a core collection of narrow-leafed lupin (111 accessions) using 191 Diversity Arrays Technology (DArT) markers. The genetic relationship among accessions was determined using the admixture model in STRUCTURE. Thirty-eight root-associated traits were characterized, with 21 having coefficient of variation values >0.5. Principal coordinate analysis and cluster analysis of the DArT markers revealed broad diversity among the accessions. An *ad hoc* statistics calculation resulted in 10 distinct populations with significant differences among and within them (*P* < 0.001). The mixed linear model test in TASSEL showed a significant association between all root traits and some DArT markers, with the numbers of markers associated with an individual trait ranging from 2 to 13. The percentage of phenotypic variation explained by any one marker ranged from 6.4 to 21.8%, with 15 associations explaining >10% of phenotypic variation. The genetic variation values ranged from 0 to 7994, with 23 associations having values >240. Root traits such as deeper roots and lateral root proliferation at depth would be useful for this species for improved adaptation to drier soil conditions. This study offers opportunities for discovering useful root traits that can be used to increase the yield of Australian cultivars across variable environmental conditions.

## Highlight

Roots in narrow-leafed lupin (*Lupinus angustifolius*) exhibit large phenotypic and genetic diversity. An association between root traits and DArT markers demonstrates potential for a marker-assisted selection programme for this species.

## Introduction

Narrow-leafed lupin (*Lupinus angustifolius* L.) has been a predominant grain legume crop and an important component of sustainable farming systems in southern Australia since its domestication was completed in Western Australia in the 1960s and 1970s (Gladstones *et al.*, 1998; Buirchell, 2008; Berger *et al.*, 2012a). It makes up about 50% of the total grain legume production in Australia, with approximately 80% produced in Western Australia (French and Buirchell, 2005; ABARES, 2014). As a legume species, narrow-leafed lupin provides substantial benefits to farming systems via the symbiotic fixation of nitrogen from the air and by acting as a disease break crop in cereal rotations. Although lupins grown in Australia are primarily used as animal feed, they also provide health benefits to humans, being gluten-free, high in protein, and low in fat and carbohydrates (Coffey, 1989; WADAF, 2007; Berger *et al.*, 2013).

Despite their economic, agricultural, and dietary importance, grain yields and planting areas of narrow-leafed lupin have declined globally in the last decade (FAO, 2013) because of low productivity and poor market value. Unlike wheat (*Triticum aestivum*), barley (*Hordeum vulgare*), and other dominant crops in Mediterranean regions, current commercial cultivars of narrow-leafed lupin incorporate only a small fraction of their genetic diversity because of the short and fragmented domestication history (Zohary, 1999; Berger *et al.*, 2013). However, the established germplasm pool of *L. angustifolius* at the Australian Lupin Collection, which comprises 2056 accessions (1327 wild, 214 cultivars, 431 advanced lines, 22 landraces, and 62 mutated lines) from diverse climatic and geographic locations, provides a broad genetic basis to improve crop breeding in this species (Clements and Cowling, 1991; Berger *et al.*, 2013). In that respect, studies on phenotypic diversity and its relationship with genetic diversity in the wild germplasm offer opportunities for discovering unexploited traits that can be used to increase the yield of Australian cultivars across a range of environmental conditions.

Diversity in root system architecture (RSA) across a substantial subsample of the world collection of narrow-leafed lupin was characterized in a recent study (Chen *et al.*, 2012). However, how the phenotypic diversity reflects the genetic diversity remains unknown. There is increasing interest in a genetic analysis of RSA and function, although the focus on the links between genes and root traits is primarily on the effects of genes that directly mediate small-scale phenomena (e.g. Little *et al.*, 2005; Bengough *et al.*, 2006, Cai *et al.*, 2012; Canè *et al.*, 2014; Mlodzinska *et al.*, 2015). One of the most effective approaches to dissecting complicated quantitative traits is an analysis of quantitative trait loci (QTL), which helps to identify specific genes responsible for trait variation (Beebe *et al.*, 2006; Weih *et al.*, 2006; Acuna *et al.*, 2014; Burton *et al.*, 2014). Several types of molecular markers, such as Diversity Arrays Technology (DArT), simple sequence repeats, amplified fragment length polymorphisms, single nucleotide polymorphisms, and sequence-tagged sites, have been developed for analysing genetic diversity in various crop species (e.g. Kwon *et al.*, 2012; El-basyoni *et al.*, 2013; Aitken *et al.*, 2014; Maccaferri *et al.*, 2015; Moore *et al.*, 2015; Uga *et al.*, 2015; Zurek *et al.*, 2015). The development of microarray hybridization-based technology, such as DArT, provides a useful tool to identify DNA variation at hundreds of genomic loci in parallel regardless of the sequence information (Jaccoud *et al.*, 2001). Array-based marker technology permits the detection of population structure and relative kinship within collections (Wenzl *et al.*, 2004; Maccaferri *et al.*, 2015). In *L. angustifolius*, DArT marker analysis revealed the low genetic diversity present in the domesticated forms (Berger *et al.*, 2012b), highlighting the need to identify and exploit useful diversity in the wild germplasm. The present study analysed genetic diversity in a core collection of wild narrow-leafed lupin using DArT markers and revealed correlations between root trait diversity (phenotype) and genetic diversity (molecular markers).

## Materials and methods

### Plant material and phenotyping

A set of 111 accessions of narrow-leafed lupin (*L. angustifolius*), consisting of 108 wild genotypes, one landrace, and two cultivars from 13 countries and four regions (Supplementary Table S1), was evaluated for phenotypic diversity in RSA traits (Table 1). They were studied under glasshouse conditions in Perth (31°58′S, 115°49′E) using a recently developed semi-hydroponic phenotyping system (Chen *et al.*, 2011a, 2012). Detailed plant growth conditions, measurements, and calculations are described in Chen *et al.* (2012). Root parameters were measured 6 weeks after planting. Taproot lengths were measured 2, 4, and 6 weeks after planting and root growth rates (RGR) were calculated and classified according to incremental increases in taproot length within a given growth period. Root subsamples were scanned in greyscale using a desktop scanner (Epson Expression 1680; Epson, CA, USA) and root images were processed in WinRHIZO v2009 Pro (Regent Instruments, QC, Canada) for root length, root surface area, volume, average root diameter, and diameter class length (DCL, root length in a diameter class). The upper 0−20cm section (separated from the plant collar) of the root system was referred to in this study as ‘topsoil’ and the lower part as ‘subsoil’. There were 38 root traits included in this study, 17 of which had not been reported previously (Chen *et al.*, 2012).

**Table 1.** Description of 38 root traits obtained in a phenotyping experiment.

**Traits**	**Description**	**Unit**	**Mean**	**Median**	**CV**	**Correlated trait No.**	**PC**
BD	Branch density (branch number/ taproot length)	m^−1^ taproot	120	120.9	0.39	31	3
BD_sub	Subsoil branch density	m^−1^	79.4	71.7	**0.50**	31	3
BD_top	Topsoil branch density	m^−1^	218.9	208.3	0.35	32	9
BI	Branch intensity (branch number/root length)	m^−1^ root	22.1	20.8	0.34	34	7
BL	Branch length	cm	343	298.7	**0.62**	26	1
BL/TRL	Branch length/taproot length		4.83	4.31	**0.53**	32	1
BL_ind	Average individual branch length	cm	4.16	3.77	0.44	28	2
BL_sub	Subsoil branch length	cm	129	100.7	**0.81**	20	1
BL_top	Topsoil branch length	cm	214	181.8	**0.62**	32	1
BLR	Branch length topsoil/subsoil ratio		2.25	1.72	**0.78**	14	7
BN	Branch number	root^−1^	84.8	78	0.47	23	1
BN_2nd	Second-order branch number	root^−1^	14.7	106	**1.20**	23	5
BN_sub	Subsoil branch number		40.8	34.8	**0.70**	24	1
BN_top	Topsoil branch number		43.8	41.7	0.35	23	9
BNR	Branch number topsoil/subsoil ratio		1.41	1.17	**0.65**	32	9
DCL_med	Root length in diameter class 0.75−1.25 mm	cm	156	134.3	**0.51**	33	1
DCL_thick	Root length in diameter class ≥1.25 mm	cm	102	89.5	**0.72**	32	1
DCL_thin	Root length in diameter class <0.75mm	cm	218	186.2	**0.64**	32	6
LBL	Length of the longest branch	cm	21.7	8	**0.90**	29	5
RA	Root surface area	cm^2^	128	112.3	**0.59**	31	1
RD	Average root diameter	mm	0.97	0.98	0.16	29	6
RGR_2−4wk	Root growth rate (2–4 weeks)	cm d^−1^	1.89	1.88	0.26	24	8
RGR_2wk	Root growth rate (0- 2 weeks)	cm d^−1^	1.92	1.86	**0.50**	23	4
RGR_4−6wk	Root growth rate (4–6 weeks)	cm d^−1^	1.61	1.51	0.24	21	6
RGR_4wk	Root growth rate (0–4 weeks)	cm d^−1^	1.72	1.77	0.23	30	2
RGR_6wk	Root growth rate (0–6 weeks)	cm d^−1^	1.32	1.29	0.44	28	3
RL	Root length	cm	416	376	**0.53**	17	1
RL_sub	Subsoil root length	cm	180	155	**0.63**	33	1
RL_top	Topsoil root length	cm	234	202	**0.57**	19	1
RLR	Root length ratio topsoil/subsoil	cm cm^−1^	1.55	1.27	**0.70**	25	7
RM	Root dry mass	mg	249	219	**0.61**	32	1
RMR	Root-to-shoot mass ratio		0.65	0.63	0.24	18	8
RTD	Root tissue density (mass/volume)	mg cm^−3^	76.9	70.6	0.45	27	9
RV	Root volume	cm^3^	3.27	2.83	**0.69**	33	1
SRL	Specific root length (length/mass)	cm mg^−1^	23.2	20.8	0.35	34	7
TRL_2wk	Taproot length after 2 weeks	cm	45.1	42.3	0.24	24	8
TRL_4wk	Taproot length after 4 weeks	cm	70.9	69.7	0.23	12	2
TRL_6wk	Taproot length	cm	26.6	26.3	0.27	22	3

Mean, median, and CV values for each trait are given. CV values >0.5 are in bold. Number of significantly correlated traits at *P* < 0.05 is given for each trait according to Pearson correlation coefficient analysis. Branch length and number refer to first-order branches unless specified. Topsoil = 0−20cm depth; subsoil = 20−120cm depth.

### DArT genotyping

A set of 191 DArT markers, including 37 mapped markers, was included in the assay (Berger *et al.*, 2012b; Kroc *et al.*, 2014). Genotyping was performed by Diversity Arrays Technology Pty Ltd (Canberra, Australia) using the protocols described by Kilian *et al.* (2012). Briefly, DNA samples of each genome were subjected to the PstI/BanII complexity reduction method (Jaccoud *et al.*, 2001). Fluorescent nucleotides were used to label the resulting genomic representations that were hybridized on a microarray printed with the DArT clones. Following hybridization and washing, the microarrays were scanned for analyses. The DArT markers were scored either ‘1’ (if a fragment present) or ‘0’ (if absent).

### Statistical analysis of trait data

IBM SPSS Statistics (Version 19, IBM Corp., Armonk, NY, USA) was used to analyse root trait data for genotype main effects with a general linear model (GLM) multivariate analysis after identifying non-significant differences between bins and harvesting times (Chen *et al.*, 2012). Descriptive statistics were computed for each trait across all genotypes in IBM SPSS Statistics 19 (IBM Corp.). The coefficient of variation (CV) was calculated by dividing SD by the mean value. Pearson correlation coefficients (*r*) were used to determine the general relationship between root trait pairs (*P* ≤ 0.05) and to generate an agglomerative hierarchical clustering (AHC) dendrogram tree. Variability in root traits across genotypes was determined by principal component analysis (PCA; Jolliffe, 2002). Rotation converged in 30 iterations using Varimax with the Kaiser Normalization method; principal components (PCs) with eigenvalues >1.0 were considered significant (Tabachnik and Fidell, 1996).

### Marker diversity analysis

The polymorphic information content (PIC) value indicates the informativeness of a marker locus or marker system. PIC was determined as follows:

$$\text{PIC}=1-\sum^{\text{​}}P_{i}^{2}$$

where $P_{i}$ is the frequency of the *i* ^th^ allele in the examined genotypes (Weir, 1990). PIC values and the marker present frequency of each DArT marker were computed in PowerMarker 3.25 (Liu and Muse, 2005). The quality parameter Q for each marker was calculated by dividing the variance of the hybridization level for the marker between the two clusters (i.e. present and absent) by the total variance of the hybridization level of the marker.

### Population structure analysis

The genetic diversity structure of the 111 genotypes was analysed using a distance-based method (Schlüter and Harris, 2006) and a model-based approach (Pritchard *et al.*, 2000). Principal coordinate analysis (PCoA) was generated using Jaccard similarity matrices in FAMD 1.25 software (Schlüter and Harris, 2006). Two-dimensional scores were calculated and used to produce scatter plot matrices of scores. Jaccard’s similarity coefficient is defined as:

$$s_{ij}=\frac{n_{11}}{n_{11}+n_{01}+n_{10}}$$

where $n_{xy}$ denotes the number of markers for which the indicated combination of character states is found for a pair of samples *i* and *j*. Character states are band presence (1), band absence (0), and missing data (?). Jaccard’s coefficient was used for the clustering analysis with the neighbour-joining (NJ) method.

Members (accessions/genotypes) in subgroups were identified using a model-based approach for dominant DArT markers implemented in the STRUCTURE software (Pritchard *et al.*, 2000). We used an admixture co-ancestry model with independent and correlated allele frequencies and a burn-in time of 50000. The number of Markov Chain Monte Carlo replications after burn-in was set at 100000 (Pritchard and Wen, 2004), with a *K* (number of populations) of up to 15 on the entire dataset (111 genotypes). The software provides the likelihood (the posterior probability) of the data for a given number of assumed populations *K*, and the value of *K* with the highest likelihood can be interpreted to correspond to an estimate for the underlying number of clusters. An *ad hoc* quantity (Δ*K*) based on the rate of the log probability of data between successive *K* values was used to determine the best *K* (Evanno *et al.*, 2005):

$$\Delta K=\frac{m\left(\left[L^{\text{''}}K\right]\right)}{S\left[L\left(K\right)\right]}$$

where L(K)=LnP(D), the log likelihood (estimated probability) for each K; $L^{'}\left(K\right)=L{\left(K\right)}_{n}-L{\left(K\right)}_{n-1};>$ $\;L^{''}\left(K\right)=L^{'}{\left(K\right)}_{n}-L^{'}{\left(K\right)}_{n-1};$ and  S[L(K)] is the SD.

Marker-based relative kinship estimates have proven useful for quantitative inheritance studies in different populations (Loiselle *et al.*, 1995; Ritland, 1996). This *K* estimate approximates identity by descent via adjusting the probability of identity by state between two individuals using the average probability of identity by state between random individuals (Yu *et al.*, 2006). Using the best *K*, STRUCTURE computed a pairwise matrix, the allele-frequency divergence (i.e. the net nucleotide distance, *δ*), which was used to construct a phylogenetic tree topology according to an NJ method (Saitou and Nei, 1987) in MEGA 2.1 (Tamura *et al.*, 2011). The analysis of molecular variance (AMOVA) was performed using standard Jaccard’s coefficients and a distance transformation (d=1−s) to identify significant differences among populations and within populations (Excoffier *et al.*, 1992).

Shannon’s index of diversity (H'), variance, and SD were calculated to measure the diversity of populations in the core collection (Shannon, 1948):

$$H^{'}=-\overset{s}{\underset{i=1}{\sum }}\frac{n_{i}}{N}ln\left(\frac{n_{i}}{N}\right)$$

where *s* is the number of populations observed, $n_{i}$ is the number observed from the *i* ^th^ population, and *N* is the total number of individuals observed in the sample. *P*-values of t-tests based on H' and variances were computed using both Bowman’s and the bootstrap (10 000 times) method (Bowman *et al.*, 1969). To further assess the existence of a genetic structure between identified clusters (populations), pairwise fixation index ($F_{st},$ or *Phi*_*st*_) values were calculated as the proportion of population variance due to among-population variation [i.e.$\;\emptyset_{\text{ST}}=\frac{\text{Va}}{\text{Va}+\text{Vb}}$] (Weir and Cockerham, 1984). The total population that was used to calculate $\emptyset_{\text{ST}}$ among Pop1 and Pop2 conformed to Pop1 + Pop2 using the software STRUCTURE and was tested by permutation. The $F_{st}$ values range from 0 to 1. A zero value implies complete panmixia (the two populations are interbreeding freely), whereas a value of 1 implies that all genetic variation is explained by the population structure (the two populations do not share any genetic diversity).

### Trait-marker association analysis

A mixed linear model (MLM) association test of root traits incorporating population structure (*Q*) and relative kinship (*K*_*r*_) matrices was performed using the TASSEL (v. 2.1) software package (Yu *et al.*, 2006; Bradbury *et al.*, 2007). We also performed GLM (Bradbury *et al.*, 2007) and structured association (SA) (Thornsberry *et al.*, 2001) analyses with the same data, incorporating population structure information as a covariate and using 1000 permutations for the correction of multiple testing. Given that the MLM method performs better in controlling spurious associations (Yu *et al.*, 2006; Aulchenko *et al.*, 2007), we first ranked significant association from the MLM (*P* ≤ 0.05) and then compared the significance of these markers (*P* ≤ 0.05) in the permutation-based GLM and SA association tests.

## Results

### Root trait variation and correlations

A total of 38 root traits, including 17 previously not described, were obtained from the phenotyping experiment (Chen *et al.*, 2012; Table 1). No serious departure from multivariate normality was found in a GLM analysis involving all trait data (the multivariate standard errors of skewness and kurtosis were 0.23 and 0.45, respectively). Values of the CV among the measured traits ranged from 0.16 (average root diameter [RD], branch density [BD]) to 1.2 (second-order branch number [BN_2nd]). Twenty-one traits had CV values >0.5 (Table 1).

**Fig. 1.**
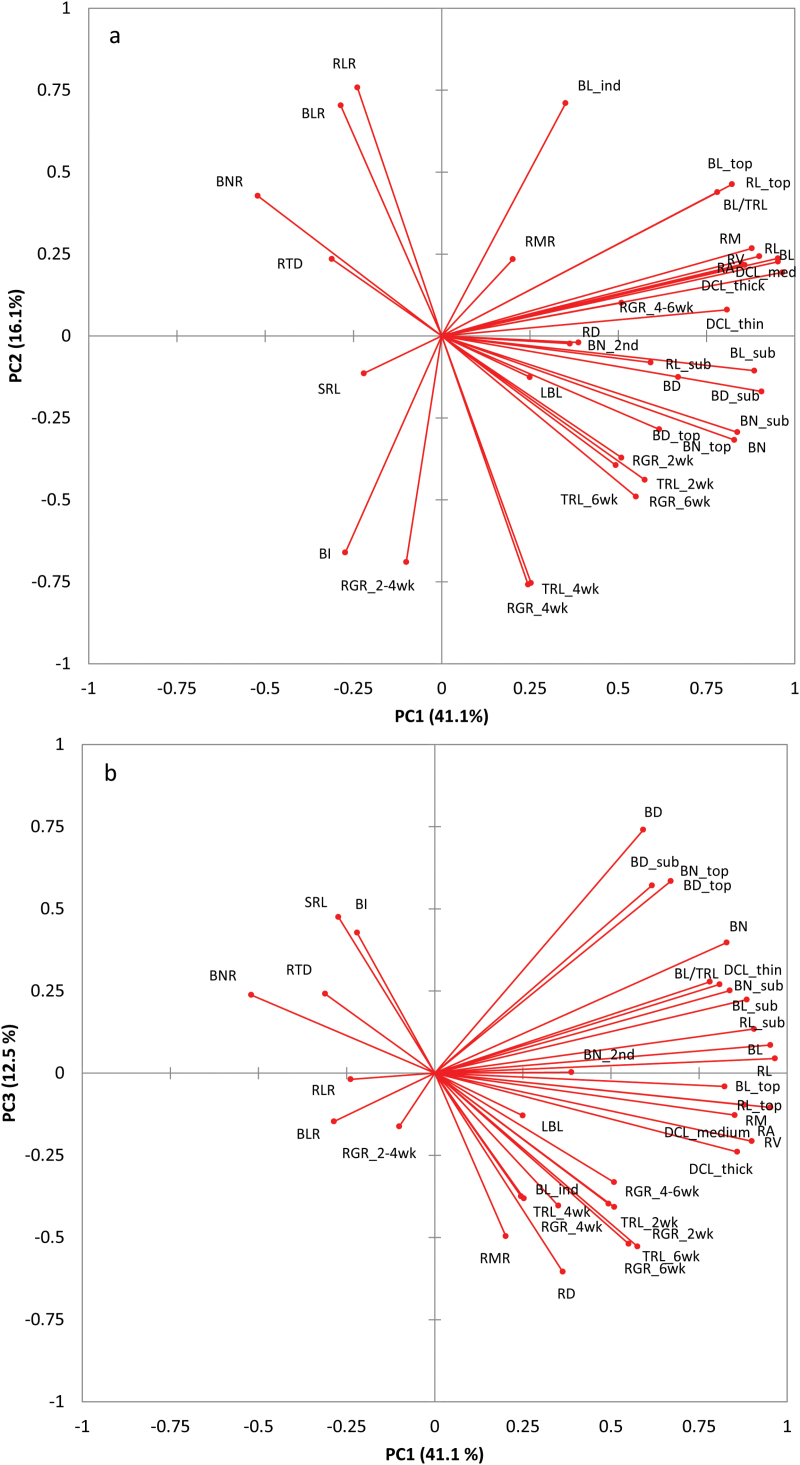
Two-dimensional plot showing the variability of 38 root traits across 111 genotypes of *L. angustifolius* based on PCA. Components PC1 versus PC2 (**a**) and PC1 versus PC3 (**b**) represent 57.2% and 53.6% of the variability, respectively. For root trait notations see Table 1. This figure is available in colour at *JXB* online.

Pearson correlation coefficient analysis on the 38 root traits showed high correlations among individual traits. The number of traits significantly correlated to an individual trait ranged from 12 to 34 at *P* ≤ 0.05 (Table 1; correlation matrix not shown). To account for these correlations, multivariate traits were constructed using PCA, resulting in nine components (PCs) with eigenvalues >1 (Supplementary Fig. S1). The number of root traits allocated to an individual PC varied from 1 to 14, with PC4 containing only root growth rate at 0–2 weeks (RGR_2wk), and PC1 having 14 root traits including branch length (BL), branch number (BN), root length (RL), and root mass (RM) (Table 1). The scree plot of the PCA exhibited the total variance explained for each component. Nine components accounted for 90.7% of the variance (Supplementary Fig. S1). Among these, the first three components (PC1, PC2, and PC3; Fig. 1) represented 41.1%, 16.1%, and 12.5% of the variance, respectively, to explain a total of 69.7% of the variance.

### Phenotypic diversity among the collection

**Fig. 2.**
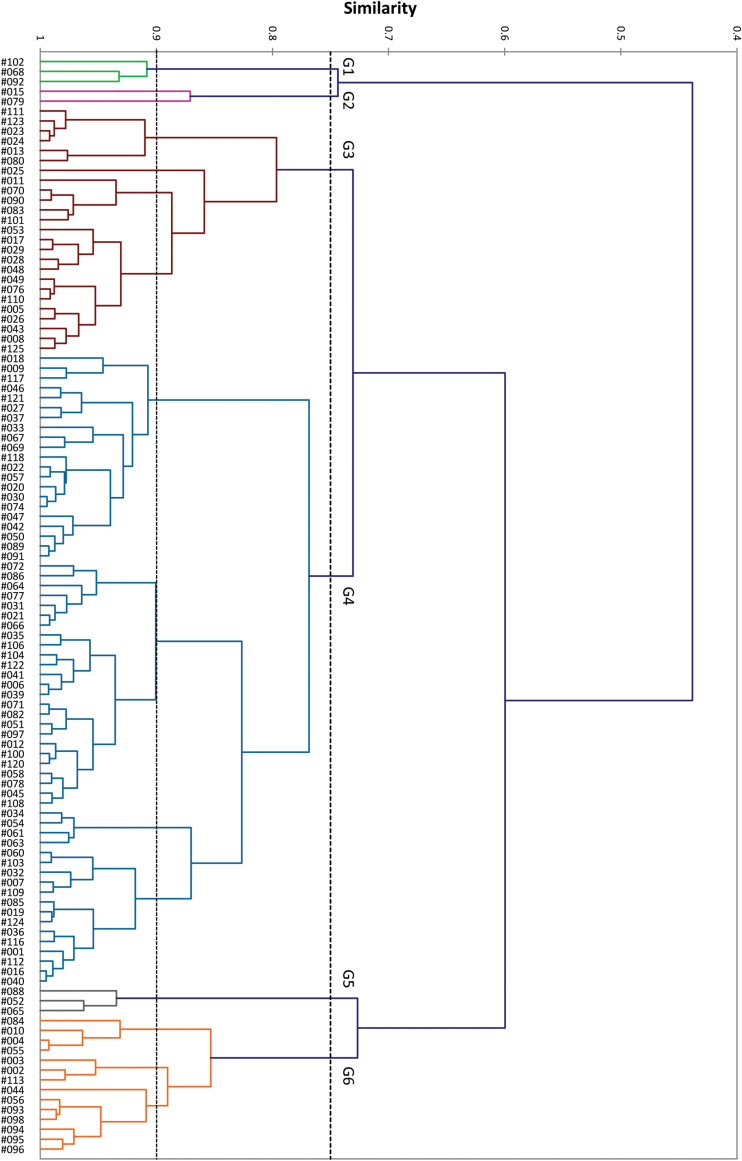
Dendrogram of AHC using the Pearson correlation coefficient on 38 root traits in XLSTAT (v2013.1). The 111 genotypes were assigned into one of six general groups (G1 to G6) at 0.75 similarity level (upper dashed line) containing 16 subgroups at 0.9 similarity level (lower dashed line). For more details on genotypes see Supplementary Table S1. This figure is available in colour at *JXB* online.

An AHC similarity dendrogram constructed with the Pearson correlation coefficients of root trait data showed a large diversity in root architecture traits among the core collection (Fig. 2). Six general groups of genotypes with relatively homogeneous root traits were identified at a similarity level of 0.75. The number of group members (genotypes) varied widely among groups. The smallest group (G2) contained two genotypes whereas the largest group (G4) consisted of 64 genotypes. At a similarity level of 0.9, groups G1, G3, G4, and G6 were further divided into two, four, five, and three subgroups, respectively. The grouping outcomes for genotypic variability and similarity in root traits did not reflect geographic origin (cf. Supplementary Table S1).

### DArT marker variation

A total of 191 DArT markers were polymorphic among the 111 accessions. The present set of DArT markers contained between 0 and 28% missing observations. The PIC values of these markers varied from 0.086 to 0.375, with an average PIC value of 0.330 (Table 2). Marker present frequency of each marker ranged from 0.11 to 0.86 with an average of 0.41 (data not shown).

**Table 2.** PIC values for 191 DArT markers used for L. angustifolius.

**PIC value** ^**a**^	**Number of DArT markers**	**% total DArT markers**
0.4−0.3	150	78.5
0.3−0.2	33	17.3
0.2−0.1	6	3.1
0.1−0.0	2	1.1

^a^ PIC values ranged from 0.086 to 0.36 with mean 0.33.

### Genetic diversity in the collection

**Fig. 3.**
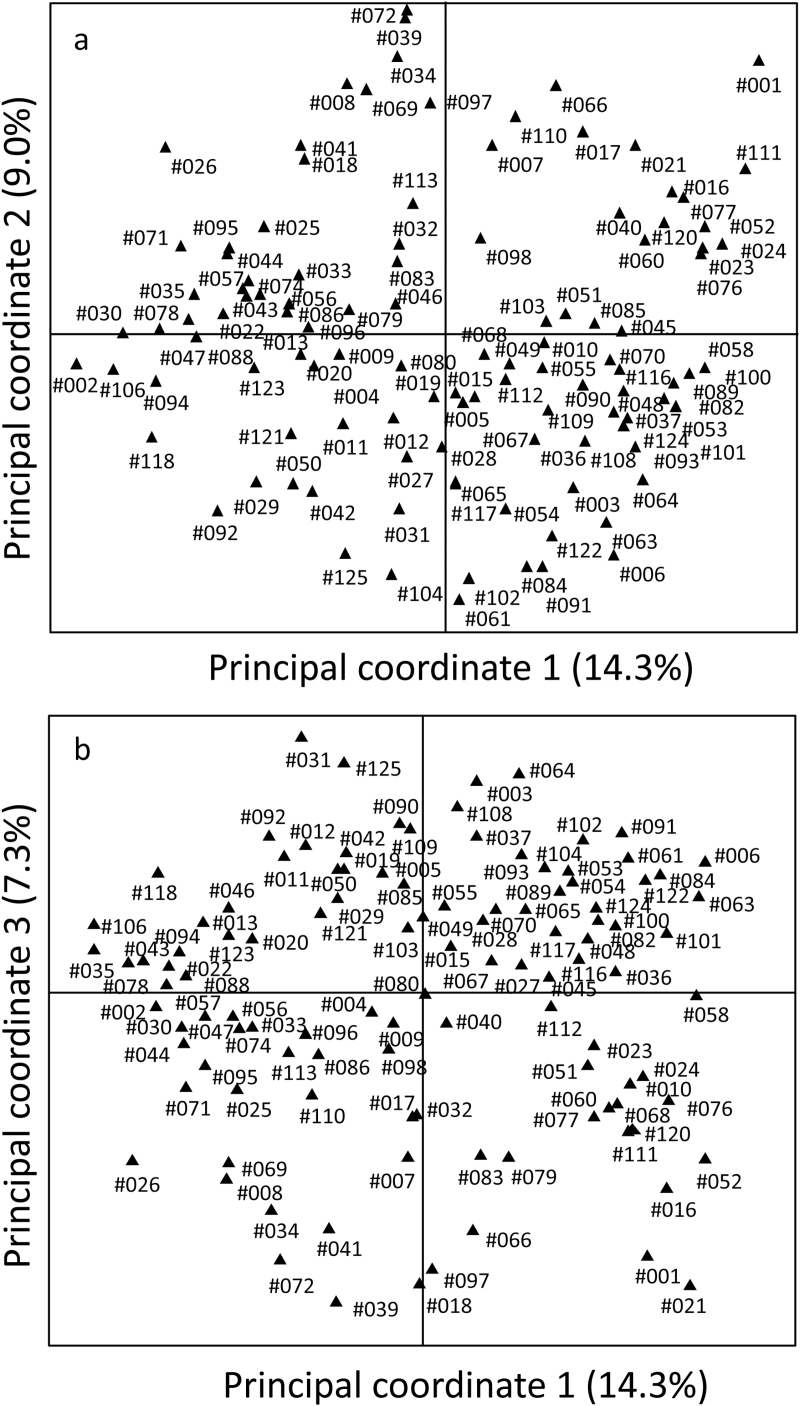
PCoA of 111 *L. angustifolius* genotypes based on 191 DArT markers. The graphs show the position of each accession in the space spanned by coordinate 1 versus coordinate 2 (**a**), and coordinate 1 versus coordinate 3 (**b**) of a relative Jaccard similarity matrix with FAMD. For root trait notations see Table 1.

**Fig. 4.**
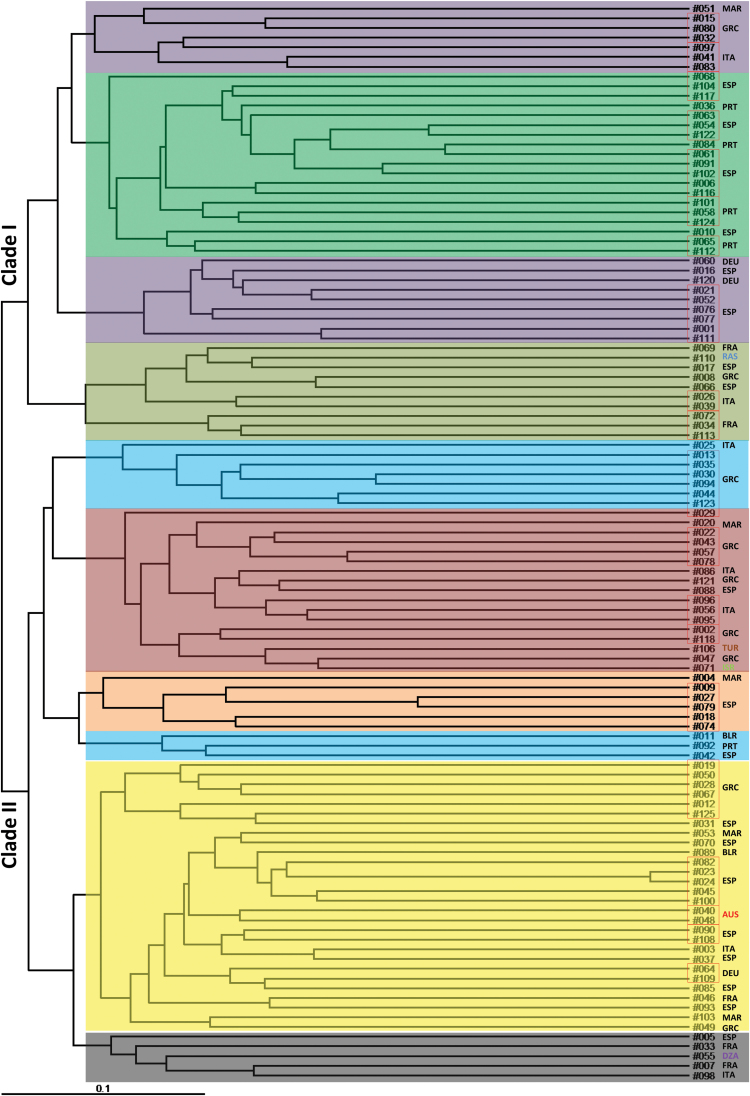
NJ tree of 111 *L. angustifolius* genotypes against 191 DArT markers with distance based on Jaccard’s coefficient. Country codes (on the right): AUS, Australia; BLR, Belarus; DEU, Germany; DZA, Algeria; ESP, Spain; FRA, France; GRC, Greece; ISR, Israel; ITA, Italy; MAR, Morocco; PRT, Portugal; RUS, Russia; TUR, Turkey. For more details on genotypes see Supplementary Table S1. This figure is available in colour at *JXB* online.

The genetic diversity of the 111 genotypes was assessed by PCoA using 191 DArT markers. PCoA identified 65 principal coordinates with positive eigenvalues, including 28 with values >1, indicating large diversity in the collection. The generated Jaccard similarity matrix was used to construct principal coordinate plots deciphering the genetic relationships among the genotypes. The first two principal coordinates derived from the scores jointly explained 23.3% of the total variance (Fig. 3). NJ tree topology constructed on the basis of the inter-individual genetic similarity (Jaccard’s coefficient) against 191 DArT markers showed a clear separation for most of the genotypes, suggesting significant diversity in this collection of accessions (Fig. 4).

### Population structure of the collection

**Fig. 5:**
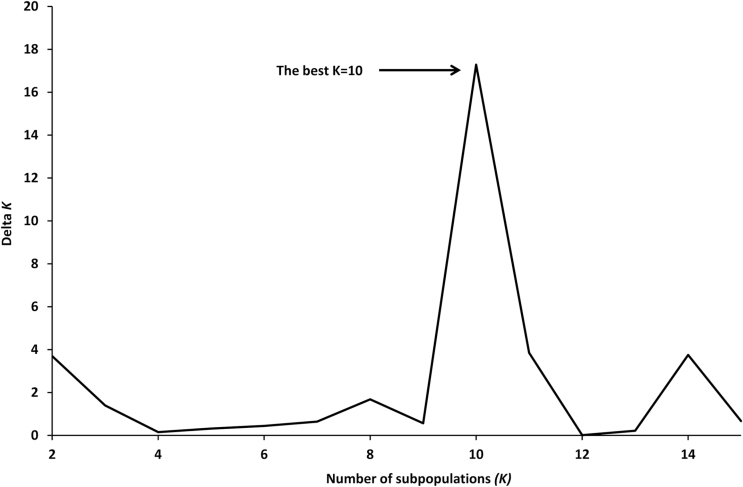
Determination of the true number of groups based on the second order rate of change of the likelihood (Δ*K*, see the calculation methods; Evanno *et al.*, 2005) using DArT marker data. The true *K*(10) is shown as the uppermost level of structure.

The genetic relationship among the 111 genotypes was analysed based on the DArT dataset. The true number of groups (populations) was determined using the admixture model in STRUCTURE and an *ad hoc* statistics (*ΔK*) calculation resulted in 10 distinct populations (Fig. 5). Differences among the 10 populations and within populations were significant based on AMOVA (*P* < 0.001) (Table 3). The genetic distances among populations were illustrated by an NJ tree using Jaccard’s coefficient (Fig. 4), and were consistent with the analysis using the allele-frequency divergence (net nucleotide distance, *δ*; data not shown). The average distance between individuals within each population ranged from 0.172 (Pop10) to 0.301 (Pop06) (Table 4). The composition of each population varied between 3 (Pop08) and 28 (Pop09) genotypes and consisted of collections from two to eight countries of origin. The two Australian cultivars in population 9 (Pop09) had a close genetic relationship with 26 other genotypes originating from Spain (12), Greece (7), Morocco (2), Germany (2), Belarus (1), France (1), and Italy (1). Forty-one genotypes from Spain were grouped into eight populations, indicating large diversity even within the same country of origin. There was no clear correlation between genetic relationship and geographic origin.

**Table 3.** AMOVA based on PCA-generated populations using standard Jaccard’s coefficients with distance transformation (d = 1− s).

**Source**	**d.f.**	**SD**	**Variance**	**Variance,** **% of total**	***P***
Among populations	9	7.57	0.066	32.55	<0.001
Within populations	101	13.9	0.138	67.45	<0.001
Total	110	21.5	0.204		

**Table 4.** Composition of 10 populations obtained by the admixture model (Q matrix in STRUCTURE) in terms of entries belonging to particular countries of origin.

**Continent**	**Country**	**Pop01**	**Pop02**	**Pop03**	**Pop04**	**Pop05**	**Pop06**	**Pop07**	**Pop08**	**Pop09**	**Pop10**	**Subtotal**
Africa	Algeria										1	**1**
	Morocco	1					1	1		2		**5**
Asia	Russia				1							**1**
	Turkey						1					**1**
Europe	Belarus								1	1		**2**
	France				4					1	2	**7**
	Germany			2						2		**4**
	Greece	3			1	6	9			7		**26**
	Israel						1					**1**
	Italy	3			2	1	4			1	1	**12**
	Portugal		7						1			**8**
	Spain		12	7	2		1	5	1	12	1	**41**
Oceania	Australia									2		**2**
**Subtotal**		**7**	**19**	**9**	**10**	**7**	**17**	**6**	**3**	**28**	**5**	111
Average distances		0.213	0.207	0.236	0.193	0.301	0.310	0.204	0.194	0.215	0.172	

### Genetic variation among populations

Shannon’s index of diversity (*H′*) and the associated variance and SD (Table 5) were computed for 10 populations generated by PCA. Both Bowman’s and bootstrap methods generated similar *H′* values for specific populations and geographic regions. However, Bowman’s method produced larger variances for each category than the bootstrap method (Table 5). Pop06 had the lowest *H′* value with the largest variance among all populations in both analyses. In contrast, Pop02 had the highest *H′* value with the smallest variance. Variations in Shannon’s index of diversity were related to the size of populations, reflecting variation in the geographic locations of each population (Tables 4 and 5).

**Table 5.** Shannon’s index of diversity (H′), variance, and SD based on 10 populations and geographic regions computed using Bowman’s and bootstrap approaches in THE FAMD package.

**Group**	**Size**	**Bowman’s method**			**Bootstrapped (10 000 times) method**		
		**Shannon’s index**	**Variance**	**SD**	**Shannon’s index**	**Variance**	**SD**
Population							
P01	7	6.91	2.74	1.66	6.92	0.005	0.068
P02	19	7.33	0.13	0.36	7.33	0.001	0.023
P03	9	7.21	0.54	0.74	7.23	0.002	0.040
P04	10	7.25	0.58	0.76	7.23	0.001	0.034
P05	7	7.26	0.77	0.88	7.25	0.001	0.037
P06	17	6.19	9.00	3.00	6.17	0.019	0.137
P07	6	7.13	1.31	1.15	7.14	0.002	0.048
P08	3	7.23	0.52	0.73	7.22	0.002	0.042
P09	28	7.25	0.64	0.80	7.26	0.001	0.036
P10	5	7.05	3.07	1.75	7.05	0.003	0.056
Region							
Africa	6	7.35	2.55	1.60	7.36	0.001	0.027
Asia	2	6.43	10.6	3.26	6.37	0.015	0.120
Europe	101	7.47	0.01	0.11	7.47	0.000	0.011
Australia	2	6.13	8.63	2.94	6.14	0.019	0.138

T-test analyses on population data exhibited significant differences between most population pairs (*P* ≤ 0.05; Table 6). For example, Pop01 significantly differed from all other populations (seven populations at *P ≤* 0.01 level and one population at *P ≤* 0.05) except for Pop10. Pop03 was significantly different from Pop01 and Pop06 (*P* < 0.01) and Pop10 (*P* ≤ 0 .05), but did not statistically differ from the other six populations. Population–population distances based on the Bayesian method ranged from 0.077 (Pop03 versus Pop05) to 0.146 (Pop02 versus Pop 03), indicating varied genetic relationships among populations (Supplementary Table S2). The genetic structure between identified populations was further assessed using pairwise *F*_*st*_ analysis. The *F*_*st*_ values estimated for population pairs ranged from 0.129 to 0.398, confirming pronounced genetic differentiation among populations (Supplementary Table S3).

**Table 6.** P values of T-test for populations (upper part) and geographic regions (lower part) computed on Shannon’s index values and variances of the 10 populations that resulted from PCA analysis using bootstrapped method.

	**Pop01**	**Pop02**	**Pop03**	**Pop04**	**Pop05**	**Pop06**	**Pop07**	**Pop08**	**Pop09**
Pop02	0.001**								
Pop03	0.004**	0.033							
Pop04	0.003**	0.026*	0.883						
Pop05	0.002**	0.069	0.708	0.800					
Pop06	0.008**	0.004**	0.005**	0.005**	0.005**				
Pop07	0.025*	0.004**	0.181	0.127	0.094*	0.007**			
Pop08	0.003**	0.033*	0.949	0.832	0.666	0.005**	0.206		
Pop09	0.002**	0.107	0.532	0.600	0.800	0.005**	0.060	0.499	
Pop10	0.162	0.003**	0.029*	0.022*	0.016*	0.010*	0.271	0.034*	0.011*

	**Africa**	**Asia**	**Europe**						
Asia	0.004**								
Europe	0.003**	0.003**							
Australia	0.003**	0.268*ns*	0.002**						

**P < 0.01; *P ≤ 0.05; ns = not significant. *P*-values obtained using Bowman’s method were all >0.05 (data not presented).

### Trait-marker association

The MLM association test of root traits revealed associations between root traits and DArT markers. All 38 root traits showed significant (*P* ≤ 0.05) associations with DArT markers, while the number of markers associated with an individual trait ranged from 2 to 13 (Table 7). At a significance level of 0.01, 30 traits were associated with one to four marker(s) (Tables 7 and 8). Of these, the branch number topsoil to subsoil ratio (BNR) was associated with four markers (lPb−328947, lPb−329087, lPb−329141, and lPb−332488) (Table 8), and average root diameter (RD) was associated with four different markers (lPb−330348, lPb−333127, lPb−333527, and lPb−334753). Thirty of the 191 markers showed a significant association with root traits (*α* = 0.01). Among them, 16 were associated with multiple traits (two to eight), whereas each of the remaining 14 was associated with a single trait. Marker IPb–333104 had the highest association with root traits, including branch density (BD), branch number (BN), subsoil branch length (BL_sub), and root length in diameter class <0.75mm (DCL_thin) (Table 8). The percentage of phenotypic variation explained by a marker (Marker *R* ^*2*^) ranged from 6.4 (branch length topsoil to subsoil ratio, BLR) to 21.8 (root tissue density, RTD), with 15 associations having Marker *R* ^*2*^ values >10%. Genetic variation values ranged from 0 to 7994, with 23 associations having values >240. A wide range of values was observed for residual variation (0−17897).

**Table 7.** Significant marker-trait associations analysis in narrow-leafed lupin.

**Traits**	**DArT marker number**	
	**α = 0.05**	**α = 0.01**
BD	7	2
BD_sub	7	1
BD_top	9	3
BI	6	1
BL	2	0
BL/TRL	2	0
BL_ind	2	0
BL_sub	13	2
BL_top	4	0
BLR	7	2
BN	11	2
BN_2nd	8	3
BN_sub	8	2
BN_top	9	3
BNR	11	4
DCL_med	11	1
DCL_thick	12	1
DCL_thin	5	3
LBL	9	1
RA	5	0
RD	12	4
RGR_2−4wk	9	1
RGR_2wk	9	3
RGR_4−6wk	9	3
RGR_4wk	10	1
RGR_6wk	12	3
RL	2	0
RL_sub	11	1
RL_top	4	0
RLR	6	0
RM	13	1
RMR	5	2
RTD	4	3
RV	7	1
SRL	5	2
TRL_2wk	9	3
TRL_4wk	8	1
TRL_6wk	9	2

**Table 8.** Significant DArT markers associated with root traits of narrow-leafed lupin.

**Trait**	**Marker**	**Lineage groups**	**Distance,** **cM**	**Site**	***F***	***P*-value**	**Error** **DF**	**Marker** ***R*** ^***2***^	**Genetic** **variance**	**Residual** **variance**	**−2Ln** **likelihood**
BD	lPb−333104			97/226	11.6	0.001	97	0.125	606	2101	1155
BD	lPb−333527	NLL−01	96.2	29	7.5	0.007	106	0.070	606	2101	1155
BD_sub	lPb−333104			97/226	7.1	0.009	97	0.080	393	1535	1120
BD_top	lPb−329031			42	7.0	0.010	94	0.068	1557	4090	1261
BD_top	lPb−333104			97/226	18.0	0.000	97	0.194	1557	4090	1261
BD_top	lPb−333527	NLL−01	96.2	29	7.5	0.007	106	0.069	1557	4090	1261
BI	lPb−333615			117/246	10.8	0.001	96	0.128	0	63	759
BL_sub	lPb−332834			89/218	10.3	0.002	106	0.096	7482	4694	1318
BL_sub	lPb−333104			97/226	7.1	0.009	97	0.069	7482	4694	1318
BLR	801605349012_H_24			178/307	6.9	0.010	106	0.064	0.9	1.9	427
BLR	lPb−329428			52	9.1	0.003	95	0.087	0.9	1.9	427
BN	lPb−329031			42	7.2	0.009	94	0.068	504	1010	1115
BN	lPb−333104			97/216	15.5	0.000	97	0.153	504	1010	1115
BN_2nd	801605349014_O_17			186/315	9.1	0.003	89	0.096	24	268	898
BN_2nd	lPb−333741			124/253	7.1	0.009	95	0.078	24	268	898
BN_2nd	lPb−334500	NLL−16	38.5	2	8.1	0.006	101	0.079	24	268	898
BN_sub	801605349003_F_3			171/300	7.4	0.008	88	0.076	278	528	1040
BN_sub	lPb−333104			97/226	11.4	0.001	97	0.111	278	528	1040
BN_top	lPb−329031			42	7.0	0.010	94	0.068	62	164	907
BN_top	lPb−333104			97/226	18.2	0.000	97	0.194	62	164	907
BN_top	lPb−333527	NLL−01	96.2	29	7.5	0.007	106	0.069	62	164	907
BNR	lPb−328947			40	7.6	0.007	107	0.070	0.5	0.7	283
BNR	lPb−329087			43	13.4	0.000	99	0.132	0.5	0.7	283
BNR	lPb−329141			45	11.1	0.001	93	0.116	0.5	0.7	283
BNR	lPb−332488			86/215	11.5	0.001	102	0.106	0.5	0.7	283
DCL_medium	lPb−334226			143/272	7.6	0.007	97	0.079	1267	5118	1268
DCL_thick	lPb−329803			57	7.9	0.006	94	0.082	1724	3850	1246
DCL_thin	lPb−333104			97/226	7.2	0.008	97	0.080	5615	17898	1392
DCL_thin	lPb−333527	NLL−01	96.2	29	7.9	0.006	106	0.074	5615	17898	1392
DCL_thin	lPb−334226			143/272	8.7	0.004	97	0.084	5615	17898	1392
LBL	801605349007_M_5			175/304	8.1	0.005	96	0.083	247	261	914.1
RD	lPb−330348			66/195	7.9	0.006	99	0.079	0	0	−123
RD	lPb−333127			21	10.7	0.002	89	0.126	0	0	−123
RD	lPb−333527	NLL−01	96.2	29	11.7	0.001	106	0.110	0	0	−123
RD	lPb−334753			165/294	8.9	0.004	95	0.089	0	0	−123
RGR	lPb−331019			70/199	7.6	0.007	96	0.079	0.1	0.1	94
RGR	lPb−334270			145/274	7.0	0.010	97	0.074	0.1	0.1	94
RGR	lPb−334297			147/276	9.8	0.002	94	0.099	0.1	0.1	94
RGR_2−4wk	lPb−329428			52	8.7	0.004	95	0.098	0.1	0.2	184
RGR_2wk	lPb−333220			103/232	8.5	0.004	106	0.080	0.1	0.2	148
RGR_2wk	lPb−333816			129/258	7.7	0.006	105	0.072	0.1	0.2	148
RGR_2wk	lPb−333836	NLL−07	29.1	14	7.5	0.008	83	0.097	0.1	0.2	148
RGR_4−6wk	lPb−329031			42	7.0	0.010	94	0.074	0.3	0.6	294
RGR_4−6wk	lPb−334226			143/272	8.9	0.004	97	0.095	0.3	0.6	294
RGR_4wk	lPb−333228			102/231	7.2	0.009	96	0.075	0.0	0.1	93
RL_sub	lPb−332834			89/218	10.6	0.002	106	0.098	7994	5529	1332
RM	lPb−329803			57	7.9	0.006	94	0.080	5947	17706	1409
RMR	lPb−334461			151/280	9.3	0.003	98	0.091	0	0	−96
RMR	lPb−334753			165/294	12.8	0.001	95	0.130	0	0	−96
RTD	lPb−329917			61	3.0	0.005	111	0.218	290	1030	1086
RTD	lPb−333220			103/232	12.5	0.001	106	0.113	290	1030	1086
RTD	lPb−333816			129/258	12.1	0.001	105	0.110	290	1030	1086
RV	lPb−334500	NLL−16	38.5	2	8.2	0.005	106	0.075	1.4	3.7	484
SRL	lPb−330348			66/195	8.5	0.004	99	0.084	51	19.2	754
SRL	lPb−334461			151/280	7.9	0.006	98	0.088	51	19.2	754
TRL	lPb−333816			129/258	7.4	0.008	105	0.074	72	153	909
TRL	lPb−334297			147/276	8.1	0.005	94	0.081	72	153	909
TRL_2wk	lPb−333220			103/232	8.4	0.005	106	0.078	9.6	37.9	739
TRL_2wk	lPb−333816			129/258	7.7	0.007	105	0.072	9.6	37.9	739
TRL_2wk	lPb−333836	NLL−07	29.1	14	7.7	0.007	83	0.096	9.6	37.9	739
TRL_4wk	lPb−333228			102/231	7.1	0.009	96	0.077	26	87.4	836

Trait-marker association was performed with an MLM model incorporating population structure (Q-matrix) and kinship (K_r_) in TASSEL 2.1. Marker R^2^ is the percentage of phenotypic variation explained by the marker. Only significant trait (α = 0.01)-trait-marker associations were included. Each trait is assigned to one of the nine PCs based on PCA with eigenvalues >1. The number of DArT markers found for each trait at α = 0.01 and 0.05 is presented.

## Discussion

A wide genetic diversity in a range of root traits was identified in a collection of narrow-leafed lupin (*L. angustifolius*) comprising 108 wild types from around the world (Table 1; Fig. 4). Exploiting the diverse genetic and adaptive resources of this species is critical for its future (Berger *et al.*, 2013) because the production of narrow-leafed lupin in Australia is hampered by terminal drought and a range of subsoil constraints (e.g. soil compaction, acidity, and aluminium toxicity; Turner and Asseng, 2005). These constraints limit root growth into deep horizons and thus restrict root access to water and nutrients (Adcock *et al.*, 2007; Chen *et al.*, 2014). Although the present study focused on characterizing genetic diversity in root traits, additional above-ground traits were measured in the phenotyping experiment. These included leaflet number, shoot height, shoot dry mass, total dry mass, the ratio of root dry mass to shoot dry mass, and the ratio of root dry mass to total dry mass (Chen *et al.*, 2012). Pearson correlation analysis revealed a strong correlation (mostly at *P* < 0.01) between 15 root traits (e.g. root length, branch length, branch number, specific root length, and root tissue density) and a number of above-ground traits (e.g. leaflet number and shoot dry weight) (Chen *et al.*, 2012).

RSA critically influences foraging and the capture of water and nutrients, and it thus determines crop productivity (Lynch, 1995). Studies have flagged root length, branching at depth, and seminal root angle as key traits likely to underpin further increases in the yield of crops such as wheat (e.g. Manschadi *et al.*, 2010). An increased capacity to take up water from deep soil horizons has been linked to increased yield potential in sugar beet (*Beta vulgaris*) (Ober *et al.*, 2005; Lynch and Wojciechowski, 2015); a similar connection was made for wheat in western and southern Australia (Wong and Asseng, 2006; Manschadi *et al.*, 2010) and rice (*Oryza sativa*; Kondo *et al.*, 1999; Kamoshita *et al.*, 2000). Recently, we observed better performance in 2 of 10 selected wild *L. angustifolius* genotypes when compared with local cultivars at a Western Australian farm with subsoil compaction (Chen *et al.*, 2014). Specifically selecting for improved root traits, such as root proliferation at depth, may result in yield increases, especially in drier soil conditions. This is particularly important because attempts to increase root density at depth using agronomic approaches (e.g. deep fertiliser placement and deep ripping) have been largely unsuccessful (e.g. Baddeley *et al.*, 2007). Therefore, it may be possible to improve the ability of lupin genotypes to adapt to subsoil constraints by selecting for proxy root traits from new and exotic germplasm sources.

The subset of the world collection of *L. angustifolius* evaluated in this study exhibited large phenotypic and genetic diversity in a range of root traits (Table 1). Genetic material from a wide latitudinal range, involving 108 wild types, was used in our study to ensure the identification of genotypic variability in various RSA traits. Large morphological diversity in relation to geographical origins has been observed previously in narrow-leafed lupin accessions from the western Mediterranean (Gladstones and Crosbie, 1979) and Aegean (Clements and Cowling, 1994) regions. Crop cultivars with proxy RSA traits may have improved desirable agronomic traits such as yield, drought tolerance, and resistance to nutrient deficiencies (Tuberosa *et al.*, 2002; Beebe *et al.*, 2006; Steele *et al.*, 2007). Developing high-throughput screening techniques for accurate and efficient phenotyping is critical for characterizing root-related traits in a wide-scale germplasm pool (De Dorlodot *et al.*, 2007). We have recently established a novel semi-hydroponic phenotyping system to determine genetic variation in intrinsic RSA in the world collection of narrow-leafed lupin. Based on the results of a glasshouse phenotyping experiment (Chen *et al.*, 2012), 10 genotypes with contrasting root characters were further examined in two different types of soils (Chen *et al.*, 2011b) and in the field (Chen *et al.*, 2014). There was relatively consistent ranking of genotypes between the two separate phenotyping experiments, and between phenotyping experiments and two different soil media in the glasshouse and the field (Chen *et al.*, 2011b, 2012, 2014). Eco-geographical studies and field phenotyping on above-ground traits have previously been evaluated (Clements and Cowling, 1994). Because root phenotypic data reported here were obtained from the phenotyping experiment under carefully controlled environmental conditions, field phenotyping of the same set of the lupin collection for root traits is required to explore the potential gene-by-environment interactions. The genotypic variability in root traits and potential traits of interest identified in our glasshouse phenotyping experiment form a basis for field study.

This study used a set of DArT markers for genetic analysis and demonstrated a high level of polymorphism and high quality as assessed by the call rate, scoring reproducibility, and PIC values of these markers (Table 2). Genetic markers with high-level polymorphism are critical for use in fingerprinting and marker-assisted selection (MAS) programmes (Smith *et al.*, 2000; Mace *et al.*, 2008). Diversity arrays have been widely used for rapid and economical genotyping to any genome or complex genomic mixtures (Jaccoud *et al.*, 2001; Akbari *et al.*, 2006). The DArT markers used in this study comprised 37 markers mapped on the genome of narrow-leafed lupin (Table 8). Marker technology is developing rapidly and future research will be able to incorporate 50 000 DArTseq markers (Matthew Nelson, unpublished data).

Our study showed significant correlations between root traits and molecular markers using genome-wide association analysis (Tables 7 and 8). These results have a potential application in the selection of suitable root traits for targeted edaphic environmental adaptation. Short regions of conserved synteny between *L. angustifolius* and two model legume species (*Medicago truncatula* and *Lotus japonicus*) have been identified (Nelson *et al.*, 2006, 2010; Kroc *et al.*, 2014), and a low-density survey sequence of the *L. angustifolius* genome was described with a small proportion of scaffolds and large-insert library clones assigned to linkage groups (Lesniewska *et al.*, 2011; Yang *et al.*, 2013). An improved reference genetic map of *L. angustifolius* comprising 1475 primarily gene-based marker loci was recently reported (Kamphuis *et al.*, 2015). The recent progress in genome mapping in narrow-leafed lupin provides useful tools for MAS and QTL cloning for RSA in wild *L. angustifolius* by exploiting genomic resources, candidate genes, and the knowledge gained from model species, particularly Arabidopsis (Sergeeva *et al.*, 2006), *M. truncatula* and *L. japonicus* (Choi *et al.*, 2004; Nelson *et al.*, 2010), rice (Horii *et al.*, 2005; Steele *et al.*, 2007), and maize (*Zea mays*) (Giuliani *et al.*, 2005). Combining phenotypic data of RSA features and genetic marker/QTL analysis will enable us to explore the inheritance of RSA traits in narrow-leafed lupin and to identify proxy traits, such as deeper roots and lateral root proliferation at depth, for enhancing adaptation to different edaphic environments, particularly drying soil conditions.

## Supplementary data

Supplementary data are available at *JXB* online.

Table S1. Breeding status and country of origin of 111 *L. angustifolius* genotypes used in this study.

Table S2. Population–population distances: chord distance from allele-frequency estimates based on the Bayesian (non-uniform prior from among-population information) method (FAMD).

Table S3. Estimates of pairwise *F*_*st*_ values for populations based on random allelic permutation testing of the DArT dataset (*P* < 0.01).

Figure S1. Scree plot of the PCA of all 38 root traits across 111 genotypes of *L. angustifolius* showing the total variance explained for each component (PC).

Supplementary Data
