# Supplementary material for: Root trait diversity, molecular marker diversity, and trait-marker associations in a core collection of Lupinus angustifolius
Source: J Exp Bot. 2016 Apr 5;67(12):3683–97. doi: 10.1093/jxb/erw127 (PMC4896361; doi:10.1093/jxb/erw127)
Supplement: Supplementary Data [file supp_67_12_3683__index.html]

Root trait diversity, molecular marker diversity, and trait-marker associations in a core collection of Lupinus angustifolius — Root trait diversity, molecular marker diversity, and trait-marker associations in a core collection of Lupinus angustifolius — Supplementary Data 

# Root trait diversity, molecular marker diversity, and trait-marker associations in a core collection of *Lupinus angustifolius*

## Supplementary Data

Data files

- supplementary\_tables\_S1\_S3\_figure\_S1.pdf - Supplementary Data
