# Supplementary material for: Root trait diversity, molecular marker diversity, and trait-marker associations in a core collection of Lupinus angustifolius
Source: J Exp Bot. 2016 Apr 5;67(12):3683–97. doi: 10.1093/jxb/erw127 (PMC4896361; doi:10.1093/jxb/erw127)
Supplement: Supplementary Data [file supp_erw127_supplementary_tables_S1_S3_figure_S1.pdf]

Supplementary data

Table S1. Breeding status and country of origin of 111 *L. angustifolius* genotypes used in this study

Accession	Collection Name	Breeding Status	Country of origin
**#001**	GS121	Wild (introduced)	Spain (EU)
**#002**	MAE15A	Wild (introduced)	Greece (EU)
**#003**	MJS009	Wild (introduced)	Italy (EU)
**#004**	GM027	Wild (introduced)	Morocco (AF)
**#005**	G84–212	Wild (introduced)	Spain (EU)
**#006**	L.O.–1709	Wild (introduced)	Spain (EU)
**#007**	GF049	Wild (introduced)	France (EU)
**#008**	MJS373	Wild (introduced)	Greece (EU)
**#009**	GS043	Wild (introduced)	Spain (EU)
**#010**	GS178	Wild (introduced)	Spain (EU)
**#011**	WLTAK–1	Wild (introduced)	Belarus (EU)
**#012**	GRC5055A	Wild (introduced)	Greece (EU)
**#013**	GRC5264A	Wild (introduced)	Greece (EU)
**#015**	MJS369	Wild (introduced)	Greece (EU)
**#016**	GS078	Wild (introduced)	Spain (EU)
**#017**	QS212	Wild (introduced)	Spain (EU)
**#018**	GS005	Wild (introduced)	Spain (EU)
**#019**	GRC5677A	Wild (introduced)	Greece (EU)
**#020**	MG112129	Wild (introduced)	Morocco (AF)
**#021**	GS008	Wild (introduced)	Spain (EU)
**#022**	GRC5677A	Wild (introduced)	Greece (EU)
**#023**	NS008	Wild (introduced)	Spain (EU)
**#024**	NS007	Wild (introduced)	Spain (EU)
**#025**	N3740	Wild (introduced)	Italy (EU)
**#026**	CD72	Wild (introduced)	Italy (EU)
**#027**	GS001	Wild (introduced)	Spain (EU)
**#028**	GRC5038A	Wild (introduced)	Greece (EU)
**#029**	MJS369	Wild (introduced)	Greece (EU)
**#030**	GRC6801A	Wild (introduced)	Greece (EU)
**#031**	MJS077	Wild (introduced)	Spain (EU)
**#032**	N3742	Wild (introduced)	Greece (EU)
**#033**	GF015	Wild (introduced)	France (EU)
**#034**	GF006	Wild (introduced)	France (EU)
**#035**	GRC5266A	Wild (introduced)	Greece (EU)
**#036**	G84–171	Wild (introduced)	Portugal (EU)
**#037**	G84–091	Wild (introduced)	Spain (EU)
**#039**	CD64	Wild (introduced)	Italy (EU)
**#040**	Unicrop	Cultivar (Australia)	Australia (OC)
**#041**	G103	Wild (introduced)	Italy (EU)
**#042**	G84–040	Wild (introduced)	Spain (EU)
**#043**	GRC5057A	Wild (introduced)	Greece (EU)
**#044**	MAE21A	Wild (introduced)	Greece (EU)
**#045**	MJS050	Wild (introduced)	Spain (EU)
**#046**	FRA6804A	Wild (introduced)	France (EU)
**#047**	GRC5054A	Wild (introduced)	Greece (EU)
**#048**	Uniwhite	Cultivar (Australia)	Australia (OC)
**#049**	MJS373	Wild (introduced)	Greece (EU)
**#050**	Santorini 1	Wild (introduced)	Greece (EU)
**#051**	GM022	Wild (introduced)	Morocco (AF)
**#052**	MJS176	Wild (introduced)	Spain (EU)
**#053**	MAR6019A	Wild (introduced)	Morocco (AF)
**#054**	L.O.–1726	Wild (introduced)	Spain (EU)
**#055**	AN08	Wild (introduced)	Algeria (AF)
**#056**	CD64	Wild (introduced)	Italy (EU)
**#057**	GRC5072A	Wild (introduced)	Greece (EU)
**#058**	GP004	Wild (introduced)	Portugal (EU)
**#060**	GL1034/81	Wild (introduced)	Germany (EU)
**#061**	L.O.–1336	Wild (introduced)	Spain (EU)
**#063**	L.O.–1719	Wild (introduced)	Spain (EU)
**#064**	GL1092/82	Wild (introduced)	Germany (EU)
**#065**	GP048	Wild (introduced)	Portugal (EU)
**#066**	GS137	Wild (introduced)	Spain (EU)
**#067**	MJS294	Wild (introduced)	Greece (EU)
**#068**	GS068	Wild (introduced)	Spain (EU)
**#069**	GF001	Wild (introduced)	France (EU)
**#070**	G84–072	Wild (introduced)	Spain (EU)
**#071**	Q048A	Wild (introduced)	Israel (EU)
**#072**	GF066	Wild (introduced)	France (EU)
**#074**	G84–018	Wild (introduced)	Spain (EU)
**#076**	GS124	Wild (introduced)	Spain (EU)
**#077**	GS148	Wild (introduced)	Spain (EU)
**#078**	GRC5063A	Wild (introduced)	Greece (EU)
**#079**	GS001	Wild (introduced)	Spain (EU)
**#080**	MJS369	Wild (introduced)	Greece (EU)
**#082**	MJS182	Wild (introduced)	Spain (EU)
**#083**	G104	Wild (introduced)	Italy (EU)
**#084**	L.O.–1756	Wild (introduced)	Portugal (EU)
**#085**	QS216	Wild (introduced)	Spain (EU)
**#086**	CD90	Wild (introduced)	Italy (EU)
**#088**	G84–007	Wild (introduced)	Spain (EU)
**#089**	BR10388	Wild (introduced)	Belarus (EU)
**#090**	G84–066	Wild (introduced)	Spain (EU)
**#091**	L.O.–1368	Wild (introduced)	Spain (EU)
**#092**	G84–141	Wild (introduced)	Portugal (EU)
**#093**	ORAM1	Wild (introduced)	Spain (EU)
**#094**	MAE33A	Wild (introduced)	Greece (EU)
**#095**	CD66	Wild (introduced)	Italy (EU)
**#096**	CD63	Wild (introduced)	Italy (EU)
**#097**	G051	Wild (introduced)	Italy (EU)
**#098**	CD90	Wild (introduced)	Italy (EU)
**#100**	G84–043	Wild (introduced)	Spain (EU)
**#101**	G84–159	Wild (introduced)	Portugal (EU)
**#102**	L.O.–1352	Wild (introduced)	Spain (EU)
**#103**	MAR6031A	Wild (introduced)	Morocco (AF)
**#104**	L.O.–1692	Wild (introduced)	Spain (EU)
**#106**	96TUR41A	Wild (introduced)	Turkey (AS)
**#108**	G84–009	Wild (introduced)	Spain (EU)
**#109**	GL1025/81	Wild (introduced)	Germany (EU)
**#110**	K1446	Landrace	Russia (AS)
**#111**	MJS176	Wild (introduced)	Spain (EU)
**#112**	GP051	Wild (introduced)	Portugal (EU)
**#113**	FRA6340A	Wild (introduced)	France (EU)
**#116**	G84–216	Wild (introduced)	Spain (EU)
**#117**	L.O.–1445	Wild (introduced)	Spain (EU)
**#118**	MJS328	Wild (introduced)	Greece (EU)
**#120**	GL1101/83	Wild (introduced)	Germany (EU)
**#121**	GRC5050A	Wild (introduced)	Greece (EU)
**#122**	L.O.–1736	Wild (introduced)	Spain (EU)
**#123**	GRC5680A	Wild (introduced)	Greece (EU)
**#124**	GP012	Wild (introduced)	Portugal (EU)
**#125**	MJS328	Wild (introduced)	Greece (EU)

Abbreviations for regions of origin: AF, Africa; AS, Asia; EU, Europe; and OC, Oceania/Australia.

Table S2. Population-population distances: chord distance from allele frequency estimates based on the Bayesian (non-uniform prior from among-population information) method (FAMD).

	Pop01	Pop02	Pop03	Pop04	Pop05	Pop06	Pop07	Pop08	Pop09
Pop02	0.096								
Pop03	0.102	0.146							
Pop04	0.107	0.138	0.094						
Pop05	0.101	0.127	0.077	0.105					
Pop06	0.091	0.091	0.107	0.097	0.089				
Pop07	0.090	0.109	0.120	0.105	0.111	0.093			
Pop08	0.122	0.131	0.115	0.113	0.101	0.084	0.135		
Pop09	0.103	0.082	0.122	0.135	0.103	0.080	0.105	0.112	
Pop10	0.110	0.106	0.137	0.118	0.129	0.091	0.104	0.104	0.104

Table S3. Estimates of pairwise F_{st} values populations based on random allelic permutation testing of DArT dataset (*P*<0.01).

	Pop01	Pop02	Pop03	Pop04	Pop05	Pop06	Pop07	Pop08	Pop09
Pop02	0.264								
Pop03	0.343	0.398							
Pop04	0.346	0.395	0.252						
Pop05	0.308	0.328	0.129	0.297					
Pop06	0.332	0.220	0.361	0.295	0.222				
Pop07	0.260	0.299	0.410	0.311	0.352	0.330			
Pop08	0.448	0.332	0.325	0.330	0.259	0.178	0.471		
Pop09	0.299	0.155	0.331	0.420	0.225	0.179	0.309	0.293	
Pop10	0.388	0.302	0.475	0.383	0.418	0.273	0.357	0.334	0.299

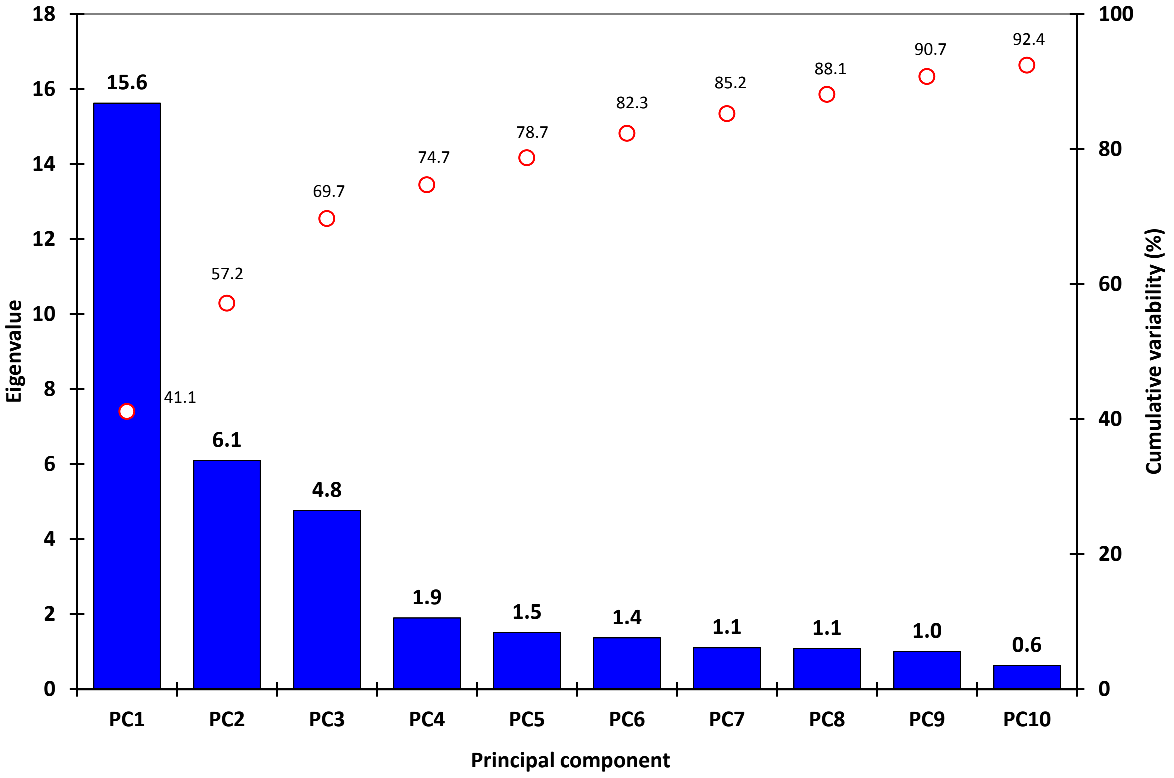

Figure S1. Scree plot of Principal Component Analysis (PCA) of all 38 root traits across 111 genotypes of *L. angustifolius* showing the total variance explained for each component (PC). Rotation converged in 30 iterations using Varimax with Kaiser Normalization method in XLSTAT (v2013.1). Eigenvalue (bars) and cumulative variability (open dots, %) are presented for the first 10 factors. PC1 to PC9 have eigenvalues greater than 1.0 and are considered significant (Tabachnik and Fidell, 1996).
